# Technology-Based Strategy to Improve Medication Compliance Among Patients With Schizophrenia Spectrum Disorders

**DOI:** 10.7759/cureus.62106

**Published:** 2024-06-10

**Authors:** Huey Jing R Tan, Shiao Ling Ling, Norashikin Khairuddin, Wan Yi Lim, Arunah Sanggar, Norliza Bt Chemi

**Affiliations:** 1 Department of Psychiatry and Mental Health, Hospital Kajang, Ministry of Health Malaysia, Kajang, MYS; 2 Department of Psychiatry, Amarantine Clinic, Kuala Lumpur, MYS; 3 Department of Psychiatry, Sungai Long Specialist Hospital, Kajang, MYS; 4 Department of Psychiatry and Mental Health, Hospital Raja Permaisuri Zainab II, Ministry of Health Malaysia, Kota Baru, MYS

**Keywords:** supermd, mobile app, non-compliance, antipsychotic medication, attitude towards psychotropic medication, telemedicine, medication adherence behaviour, schizophrenia, medication compliance

## Abstract

Introduction:* *Non-compliance to medications remains a challenging problem in schizophrenia. Newer strategies with high feasibility and acceptability are always being researched. This study aimed to assess the effectiveness of technology-based intervention in improving medication compliance in individuals with schizophrenia.

Method: This was a prospective intervention study where participants were required to use the SuperMD smartphone application (Digital-Health Technologies Pte Ltd, Kuala Lumpur, Malaysia) for a month. A change in the Medication Adherence Rating Scale-Malay Translation (MARS-M) and Malay Translation of Drug Adherence Inventory-9 (MDAI-9) scores indicated a change in compliance and attitude to medication. Positive and Negative Syndrome Scale (PANSS) was used to assess change in symptoms and insight. Medication compliance was also obtained from the SuperMD application*. *Paired T-test was used to evaluate the significance of changes in mean scores of research variables over the study period. Wilcoxon signed-rank test was used to analyze the subscale of MDAI-9 and the change in PANSS score. The Kruskal-Wallis test was used to determine the effect of the change of insight on the level of compliance with medication.

Results: There were 36 participants in this study. The results showed statistically significant improvement in compliance (0.65, p ≤ 0.01) but not in attitude towards medication (0.78, p = 0.065). There was also an improvement in PANNS score (-2.58, P ≤ 0.01). There was no significant change in insight (χ^2^(2) = 3.802, p = 0.15)*. *

Conclusion:The use of technology-based strategies like SuperMD is effective in improving medication compliance for individuals with schizophrenia.

## Introduction

Schizophrenia is a severe mental illness and antipsychotic medication remains the mainstay of treatment [1]. Many studies have found that medication non-compliance among patients with schizophrenia is common and has been widely recognized as a challenging problem in its management [2-4]. Research findings showed that the prevalence rate of medication non-compliance is 41-61% [5-7]. Stange et al. found that non-compliance was lower during hospital stay than post discharge (37.6% versus 61.2%) [8].

Among the most common reasons for non-compliance is forgetting to take medication [9,10]. Other factors that may influence medication compliance include insight, symptom severity, social support, and employment status [9-11]. Medication adherence is higher in patients on atypical antipsychotics [9,12].

Non-compliance to medication for the treatment of schizophrenia often results in frequent relapse and hospitalization, increasing healthcare cost and burden. Many strategies have been implemented to overcome problems with non-compliance among patients with schizophrenia. The interventions used in clinical settings can be broadly divided into educational, behavioral, family-based, technology-based, or a combination of the previous types [13]. Examples of these strategies include the use of intramuscular depot antipsychotic medications, community psychiatry approach, pill count, and psychoeducation [3,14-17]. Research showed that incorporating cognitive behavioral therapy and motivational interviews into clinical practice can enhance medication compliance [18]. A combination of behavioral and educational approaches is more successful in improving compliance [13].

In developing countries, often only case management-based mental healthcare teams are available for community psychiatry services [4]. Supervised treatment in outpatient for schizophrenia (STOPS), which involves a key relative of the patient in collecting, administrating, and supervising medication, was started in low and middle-income countries. A study on the efficacy of STOPS showed medication compliance was better with STOPS as compared to control groups (67.3% versus 45.5%) [19] Telephone interventions are also used to enhance medication compliance to reduce relapse and hospitalization costs [20].

With advancements in technology, newer strategies are now used to monitor and improve medication adherence. Electronic monitoring systems have been shown to demonstrate high feasibility and acceptability among patients with schizophrenia and improved compliance [21-23]. This study aimed to assess the efficacy of technology-based interventions in improving medication adherence in patients with schizophrenia.

## Materials and methods

Study design

This was a prospective intervention study conducted at Hospital Kajang, Malaysia. Adult patients aged 18-60 years and diagnosed with either brief psychotic disorder, schizophreniform, schizophrenia, or schizoaffective disorder based on the diagnostic criteria of Diagnostic Statistical Manual 5 were included. Participants with neurocognitive disorders, intellectual disability, and aggressive or suicidal behavior were excluded from the study. Participants who did not have access or did not know how to operate smartphones were also excluded from the study. Consent was obtained from all participants prior to participation in the study. The study was approved by the Ethics Committee of the National Medical Research Registry (approval number: NMRR ID-22-02648-OHA).

Sample size

Sample size estimation was done using a two-population means formula. Prior data indicate that the mean MARS score prior to intervention was 4.5 (SD=1.10) and the mean post intervention was 7.8 (SD=1.16) [24]. Thus, a minimum sample size of 1 is sufficient to reject the null hypothesis with probability (power) 0.8. The type I error probability associated with this test of null hypothesis is 0.05. Considering a 20% dropout rate, the sample size was kept at 2. 

Research instrument

Medication compliance was assessed using the Malay Translation of Drug Adherence Inventory-9 (MDAI-9) and the Medication Adherence Rating Scale-Malay Translation (MARS-M). For the purpose of this study, compliance refers to the extent to which the patient conforms to the prescribed timing, dosage, and frequency of the medication regime and is defined via an improvement in the MDAI-9 and MARS-M scores [25,26].

The MARS-M is a seven-item self-rated questionnaire validated to assess compliance in psychoses. It is a reliable and validated tool to assess medication compliance. The subscale of MARS-M has an internal consistency ranging from 0.78 to 0.84. The higher the sum score of MARS-M, the more compliant the participant is with the medication prescribed [27].

MDAI-9 is a nine-item self-rated questionnaire. It is a valid and reliable tool with good internal consistency (α = 0.7). The items on the original DAI-9 were constructed based on the understanding that compliance was influenced by the patients’ positive and negative attitudes toward the medication [28]. A positive sum score < 11 is defined as a negative attitude to medication while a negative sum score >7 suggests a side effect problem [28].

Intervention

Participants recruited were required to use the SuperMD smartphone application (Digital-Health Technologies Pte Ltd, Kuala Lumpur, Malaysia) for a month. The SuperMD application delivers automatic reminders to participants to take medication. Participants are required to click on the application within 15 minutes after the medication is taken. It also alerts the participants when medication is running low and sends out reminders for follow-up appointments.

Data collection process

Participants of this study were recruited via systematic random sampling. Demographic data was obtained from the participants’ medical records. Participants were required to fill in MARS-M and MDAI-9 before the implementation of the intervention and one month after the intervention. Positive and Negative Syndrome Scale (PANSS) was filled out by investigators before and one month after the intervention.

Following recruitment, investigators keyed in medication type, dosage, and time for medication intake into the SuperMD application. Participants were required to click "done" in the smartphone application within 15 minutes after taking medications. They were required to attend follow-up appointments one month following the intervention. Data on the number of days of medication compliance was also obtained from the SuperMD smartphone application. A green circle appeared in the application if the participant had taken the medication within the prescribed time frame. A black circle appeared in the application if the participant was not compliant with the medication. Participants were not able to alter the color of the circle in the application. If the participant did not click on the SuperMD application, it was considered as "did not take medication". For the purpose of this study, compliance was defined as taking prescribed medication ≥ 24 days in a month. Partial compliance was defined as taking prescribed medication 7-23 days in a month. Non-compliance was defined as taking prescribed medication less than seven days in a month.

Data analysis

The sum score of MARS-M and MDAI-9 was calculated. The post-test mean scores of the research variables were compared with the mean score at the pre-test. A paired T-test was used to evaluate the significance of changes in mean scores of research variables over the study period. Any change in the mean score for each section of the questionnaire, with statistical significance at 5%, was considered a change in compliance and attitude, respectively. Wilcoxon signed-rank test was used to analyze the subscale of MDAI-9 and the change in PANSS score. Kruskal-Wallis test was conducted to determine whether there was an effect in the change of insight on the level of compliance to medication. Non-parametric tests are used because normality testing showed data were not normally distributed. 

## Results

Demographics

There were 36 participants in this study. Of these, 41.7% (n = 15) were male and 58.4% (n = 21) were female. The majority of the participants (30.7%; n=11) were in the age group of 21-30 years followed by 27.9% (n=10) in the age group of 31-40 years and 22.4% (n=8) in the age group of 51-60 years. The mean age for participants was 38.39 (SD=11.46) years, and the median age was 37 years. Thirteen participants (38.9%) were married, 18 (50%18) were single, and four (11.2%) were separated, divorced, unmarried, or widowed. The summary of baseline demographic data is presented in Table 1. 

**Table 1:** Demographic characteristics of participants (N=36)

Characteristics	Number of participants	Percentage
Age		
18-20 years	1	2.8
21-30 years	11	30.7
31-40 years	10	27.9
41-50 years	6	16.8
51-60 years	8	22.4
Gender		
Male	15	41.7
Female	21	58.4
Marital Status		
Single	18	50.00
Married	13	38.9
Separated	2	5.6
Divorce	1	2.8
Widowed	1	2.8
Number of Children		
0	21	58.3
1	5	13.9
2	4	11.1
3	4	11.1
4	2	5.6
Highest Education		
Primary	1	2.8
Secondary	20	55.6
Diploma	6	16.7
Degree	7	19.4
Others	2	5.6
Household Income (Ringgit Malaysia)		
<1000	6	16.7
1000-3999	19	52.8
4000-7999	9	25.0
8000-9999	2	5.6
Occupation		
Professional	3	8.3
Teacher	1	2.8
Businessman	1	2.8
Labourer	2	5.6
Homemaker	5	13.9
Retired	2	5.6
Unemployed	12	33.3
Others	10	27.8

Change in compliance and attitude toward medication

The result of the study showed significant improvement in medication compliance as indicated via improvement in MARS-M score. The mean difference in MARS-M score was 0.64 (p = 0.001). This is accompanied by statistically significant improvement in the PANSS score. The mean difference for the PANSS score was -2.58 (p = 0.003). The mean difference in the MDAI-9 score for attitude to medication was 0.78 (p = 0.065) and for side effects, it was -0.06 (p = 0.12). Table 2 summarizes the pairwise comparison of compliance, attitude, and PANSS scores before and after the intervention. Of the participants, 44.4% (n=16) perceived that they were given autonomy in treatment decisions while 2.8% (n=1) did not perceive that they were given autonomy in treatment decisions. Perceived autonomy in treatment decisions of participants is presented in Table 3.

**Table 2:** Pairwise comparison of compliance, attitude (MARS-M and MDAI-9), and PANSS scores *p<0.05; **p<0.01 MARS-M: Medication Adherence Rating Scale-Malay Translation; MDAI-9: Malay Translation of Drug Adherence Inventory-9; PANSS: Positive and Negative Syndrome Scale

		Pretest (I)	Post test (J)	Mean Difference (J-I)	Standard Error	P-value
MARS-M		5.47	6.11	0.64	0.179	0.001**
MDAI-9	Attitude to medication	15.36	16.14	0.78	0.408	0.065
	Side effect	5.83	5.22	-0.61	2.384	0.120
PANSS	Sum Score	36.89	34.31	-2.58	9.959	0.003*

**Table 3:** Perceived autonomy in treatment decision (MDAI-9 subscale) MDAI-9: Malay Translation of Drug Adherence Inventory-9

I take medication on my free choice	Number of participants	Percentage
Agree fully to the statement	16	44.4
Agree to large extent	14	38.9
Agree to some extent	5	13.9
Do not agree	1	2.8

From the records of the SuperMD mobile application, 61.1% (n=22) of the participants were compliant ≥ 24 days in a month, 2.78% (n=1) were compliant for ≥7 days in a month, and 36.11% (n=13) were compliant for less than seven days in a month. Nineteen (52.78%) participants failed to click on the mobile application after they took the medication. Table 4 illustrates the reasons for non-compliance on the SuperMD mobile application. 

**Table 4:** Reasons for non-compliance to mobile application reminders

Reasons	Number of participants	Percentage
Forget to Click on the Application	12	63.16
Want to Stop Medication	1	5.26
Did not Hear the Reminder	1	5.26
Came Home Late	1	5.26
Did Not Want to Use the Application	1	5.26
Was Unable to Access the Application	3	15.79

Insight

Insight of participants to illness and treatment was identified via item assessing judgment and insight in PANSS. Thirty (83.3%) participants were found to have good judgment and insight during recruitment, 13.9% (n=5) of the participants were rated as having minimal or mild lack of sight before the intervention, 2.8% (n=1) of participants were moderately severe in the lack of judgment and insight during recruitment, 88.9% (n=32) of participants were noted to have good insight following the intervention, and 11.1% (n=4) of the participants had minimal or mild lack of judgment and insight post the intervention. There were no participants who had a moderately severe lack of insight post the intervention. The Kruskal-Wallis test was conducted to determine whether the change in insight at different levels of compliance to medication post intervention was significant. The results indicated a non-significant difference, χ^2^(2) = 3.802, p = 0.15. Findings are summarized in Table 5.

**Table 5:** Change in insight pre and post intervention

Insight	Pre-intervention, n (%)	Post-intervention, n (%)
Good	30 (83.3)	32 (88.9)
Mild Lack of Insight	5 (13.9)	4 (11.1)
Moderately Severe Lack of Insight	1 (2.8)	0

## Discussion

Research on the use of mobile applications to monitor medication compliance has shown promising results. Many studies have indicated that mobile applications with automatic reminders and tracking features help users stay organized and reduce missing medications [22-24]. The current study yielded results that are consistent with findings from other studies in this area and showed significant improvement in symptoms of schizophrenia spectrum disorders following improvement in medication compliance. This finding is consistent with other studies that have also highlighted the importance of good medication compliance for cost-effective treatment and relapse prevention [14]. In our study we observed that there were many participants who found the mobile application to be helpful and continued to use it after the study.

It was observed in this study that the level of insight of participants may play a role in their willingness to participate in interventions that improve medication compliance. This result aligned with many studies that have consistently indicated higher levels of insight are generally associated with better medication compliance among individuals with schizophrenia [9,11]. Of the participants, 83.3% were found to have good insight during recruitment. This may be because individuals with poor insight did not see the need to take medication or use the smartphone application and, hence, did not participate in the study. This also explained a non-statistically significant change in insight post intervention as most participants already had good insight during recruitment.

While technology-based strategies can offer significant benefits in managing schizophrenia spectrum disorders, there are limitations to consider. One key limitation is that digital interventions may not be accessible to individuals who do not have a smartphone or who may struggle with using digital devices. This could create disparities in care and support for patients with schizophrenia who are already vulnerable. In this study, we found that many participants (46.3%, n=31) were excluded from the study because they did not own mobile phones. This may be related to high rates of unemployment which often correlates to financial instability, making it difficult for them to afford necessities such as a phone and internet [29].

More than half of the participants in this study reported forgetting to click on the mobile application. Adherence to digital intervention has been found to range from medium to low in some studies [30]. Forgetfulness often emerges as the predominant factor contributing to non-compliance with mobile application reminders among individuals with schizophrenia spectrum disorders [31]. This issue reflects the cognitive challenges commonly associated with the condition. Cognitive impairment in schizophrenia spectrum disorders affects various aspects of cognitive functioning such as attention, memory, executive function, and problem-solving skills. In addition, some individuals with schizophrenia spectrum disorders may have limited exposure to technology causing them to lack the skills needed to use smartphones effectively. This can result in difficulties with basic phone functions, downloading, and using applications required for monitoring compliance. Patients who are in relapse may not be able to respond accordingly to digital reminders as well. Therefore, while technology can be a valuable tool in compliance strategies for schizophrenia, the effectiveness of mobile applications can be hindered when users struggle to recall or execute tasks due to cognitive impairment, and it is important to recognize and address these limitations to ensure holistic and effective care for individuals with this condition.

Challenges in implementing technology-based interventions in patient care also include resistance to change by staff due to fear of increased workload during the transition. Implementing these technology-based interventions during busy clinics may also cause temporary disruption of existing workflow. Therefore, smart applications should be user friendly simplifying steps for download and installation. Staff may also require comprehensive training that is resource-intensive to address skill gaps in computer literacy in order to effectively assist patients in using technology-based devices. Technology-based strategies require adequate equipment such as computers and the Internet that supports the use of digital interventions. Many public healthcare facilities in developing countries are lacking in this to support the seamless use of these interventions.

Using technology-based strategies such as SuperMD in schizophrenia spectrum disorders has been proven to enhance compliance with medication and empower patients in the treatment process. It also allows healthcare providers to remotely monitor patients’ compliance. However, as noted in this study, insight plays an important role in medication compliance in schizophrenia spectrum disorders. Therefore, incorporating strategies to improve insight-related factors such as psychoeducation or motivational interviews along with technology-based interventions is crucial in ensuring efficacy in these strategies in improving medication compliance.

Cognitive impairment is a common feature of schizophrenia spectrum disorders [31]. Addressing forgetfulness as a key barrier and tailoring digital interventions to meet the needs of individuals with schizophrenia spectrum disorders will be helpful in improving overall compliance. This will include adding features within the mobile applications to enhance memory cues for users.

Digital interventions require computers and the internet available in the healthcare facility along with staff who are technology savvy. In order to ensure the smooth implementation of these technology-based interventions, a comprehensive approach that includes stakeholder buy-in, thorough planning, effective training, ongoing support, and a focus on data security and privacy is essential.

Lastly, technology-based strategies should not replace traditional approaches. Collaborative efforts between healthcare professionals, patients, and caregivers are still important and should be integrated into these technology-based interventions in the treatment plan.

Limitations of the study

One limitation was the short duration of the study, whereby change in compliance was measured after one month of intervention. This might not be sufficient to assess the sustainability of the change in compliance and the impact on attitudes to medication. This study was done without a control group to provide a definitive conclusion on the impact of the mobile application. Subsequent outcome studies should be done with control groups and longer study duration for a more comprehensive assessment of the effectiveness of this mobile application in improving compliance and attitude towards medication.

## Conclusions

Technology-based interventions can improve medication compliance and enhance patient outcomes. It also revolutionizes healthcare delivery in the treatment of schizophrenia. However, it requires consideration of accessibility, usability, and integration with existing care systems to maximize effectiveness.

## References

[1] Skarsholm H, Stoevring H, Nielsen B (2014). Effect of a system-oriented intervention on compliance problems in schizophrenia: a pragmatic controlled trial. Schizophr Res Treatment.

[2] Higashi K, Medic G, Littlewood KJ, Diez T, Granström O, De Hert M (2013). Medication adherence in schizophrenia: factors influencing adherence and consequences of nonadherence, a systematic literature review. Ther Adv Psychopharmacol.

[3] Serobatse MB, Du Plessis E, Koen MP (2014). Interventions to promote psychiatric patients’ compliance to mental health treatment: a systematic review. Health SA Gesondheid.

[4] Tan HJ (2017). When all else fails: supervised treatment in outpatient for schizophrenia (STOPS). BMJ Case Rep.

[5] Gray R, Bressington D, Ivanecka A (2016). Is adherence therapy an effective adjunct treatment for patients with schizophrenia spectrum disorders? A systematic review and meta-analysis. BMC Psychiatry.

[6] Lacro JP, Dunn LB, Dolder CR, Leckband SG, Jeste DV (2002). Prevalence of and risk factors for medication nonadherence in patients with schizophrenia: a comprehensive review of recent literature. J Clin Psychiatry.

[7] Valenstein M, Ganoczy D, McCarthy JF, Myra Kim H, Lee TA, Blow FC (2006). Antipsychotic adherence over time among patients receiving treatment for schizophrenia: a retrospective review. J Clin Psychiatry.

[8] Stange D, Kriston L, von Wolff A, Baehr M, Dartsch DC (2013). Medication complexity, prescription behaviour and patient adherence at the interface between ambulatory and stationary medical care. Eur J Clin Pharmacol.

[9] Stentzel U, van den Berg N, Schulze LN (2018). Predictors of medication adherence among patients with severe psychiatric disorders: findings from the baseline assessment of a randomized controlled trial (Tecla). BMC Psychiatry.

[10] Mahler C, Jank S, Hermann K, Haefeli WE, Szecsenyi J (2009). Information on medications - how do chronically ill patients assess counselling on drugs in general practice? [Article in German]. Dtsch Med Wochenschr.

[11] Semahegn A, Torpey K, Manu A, Assefa N, Tesfaye G, Ankomah A (2020). Psychotropic medication non-adherence and its associated factors among patients with major psychiatric disorders: a systematic review and meta-analysis. Syst Rev.

[12] Dolder CR, Lacro JP, Dunn LB, Jeste DV (2002). Antipsychotic medication adherence: is there a difference between typical and atypical agents?. Am J Psychiatry.

[13] Loots E, Goossens E, Vanwesemael T, Morrens M, Van Rompaey B, Dilles T (2021). Interventions to improve medication adherence in patients with schizophrenia or bipolar disorders: a systematic review and meta-analysis. Int J Environ Res Public Health.

[14] García-Pérez L, Linertová R, Serrano-Pérez P, Trujillo-Martín M, Rodríguez-Rodríguez L, Valcárcel-Nazco C, Del Pino-Sedeño T (2020). Interventions to improve medication adherence in mental health: the update of a systematic review of cost-effectiveness. Int J Psychiatry Clin Pract.

[15] Torres-Robles A, Benrimoj SI, Gastelurrutia MA (2022). Effectiveness of a medication adherence management intervention in a community pharmacy setting: a cluster randomised controlled trial. BMJ Qual Saf.

[16] Tsikada OS (2020). Evidence-Based Strategies for Improving Medication Adherence Among Psychiatric Patients: A Systematic Review [Thesis]. https://scholarworks.waldenu.edu/dissertations/8811/.

[17] Steinkamp JM, Goldblatt N, Borodovsky JT, LaVertu A, Kronish IM, Marsch LA, Schuman-Olivier Z (2019). Technological interventions for medication adherence in adult mental health and substance use disorders: a systematic review. JMIR Ment Health.

[18] Inwanna S, Duangchan C, Matthews AK (2022). Effectiveness of interventions to promote medication adherence in schizophrenic populations in Thailand: a systematic review. Int J Environ Res Public Health.

[19] Farooq S, Nazar Z, Irfan M, Akhter J, Gul E, Irfan U, Naeem F (2011). Schizophrenia medication adherence in a resource-poor setting: randomised controlled trial of supervised treatment in out-patients for schizophrenia (STOPS). Br J Psychiatry.

[20] Fulmer TT, Feldman PH, Kim TS, Carty B, Beers M, Molina M, Putnam M (1999). An intervention study to enhance medication compliance in community-dwelling elderly individuals. J Gerontol Nurs.

[21] Tessier A, Dupuy M, Baylé FJ (2020). Brief interventions for improving adherence in schizophrenia: a pilot study using electronic medication event monitoring. Psychiatry Res.

[22] Velligan DI, Wang M, Diamond P (2007). Relationships among subjective and objective measures of adherence to oral antipsychotic medications. Psychiatr Serv.

[23] Kreyenbuhl J, Record EJ, Himelhoch S (2019). Development and feasibility testing of a smartphone intervention to improve adherence to antipsychotic medications. Clin Schizophr Relat Psychoses.

[24] Ibeh J (2021). Increasing Adherence to Antipsychotic Medication in Patients with Schizophrenia Using Mobile App Reminder [Thesis]. https://www.proquest.com/openview/fda0e8214365fcec226a6049c297d8d8/1?pq-origsite=gscholar&cbl=18750&diss=y.

[25] Cramer JA, Roy A, Burrell A, Fairchild CJ, Fuldeore MJ, Ollendorf DA, Wong PK (2008). Medication compliance and persistence: terminology and definitions. Value Health.

[26] Anghel LA, Farcas AM, Oprean RN (2019). An overview of the common methods used to measure treatment adherence. Med Pharm Rep.

[27] Tan HJ, Ling SL, Khairuddin N, Danaee M, Sanggar A, Lim WY, Chemi NB (2024). Psychometric properties of the Malay translation of the medication adherence rating scale. Cureus.

[28] Tan HJ, Ling SL, Khairuddin N, Sanggar A, Lim WY, Danaee M, Chemi NB (2024). Validation of Malay translation of drug attitude inventory. Cureus.

[29] Bouwmans C, de Sonneville C, Mulder CL, Hakkaart-van Roijen L (2015). Employment and the associated impact on quality of life in people diagnosed with schizophrenia. Neuropsychiatr Dis Treat.

[30] Lim MH, Penn DL (2018). Using digital technology in the treatment of schizophrenia. Schizophr Bull.

[31] Bowie CR, Harvey PD (2006). Cognitive deficits and functional outcome in schizophrenia. Neuropsychiatr Dis Treat.

